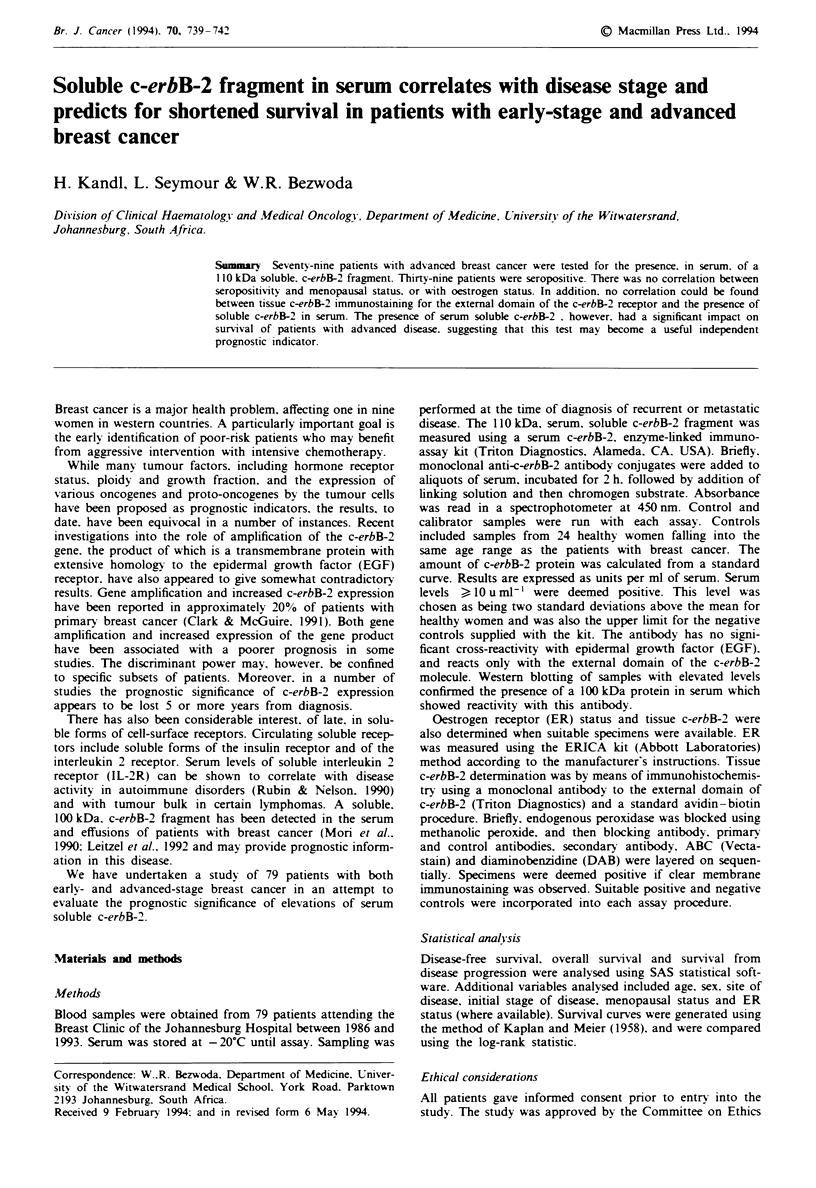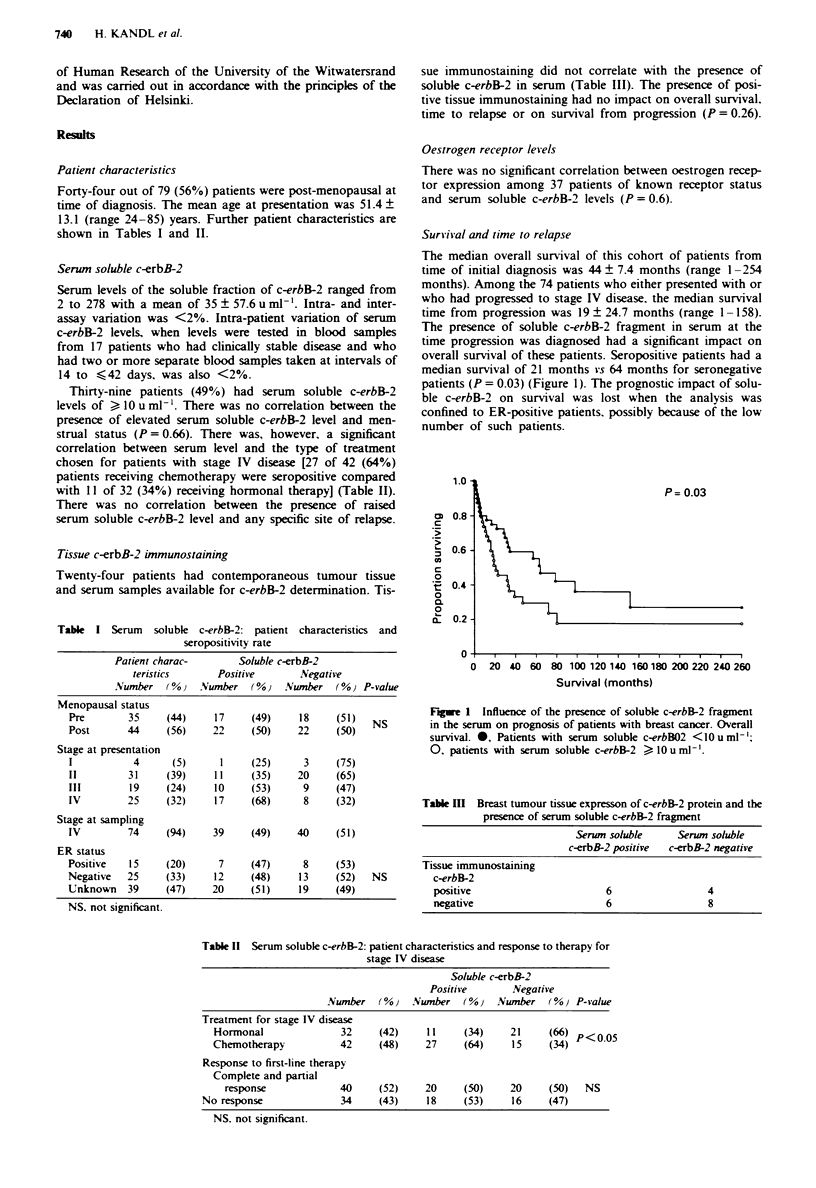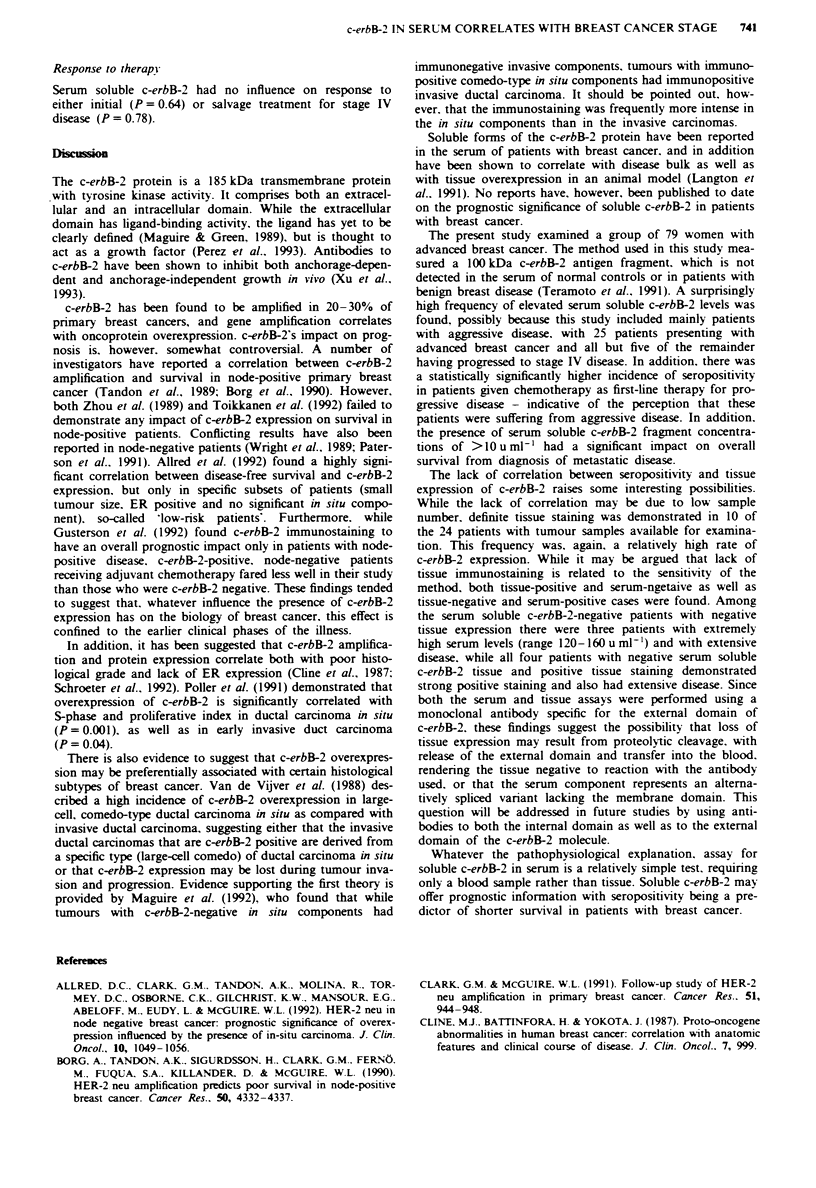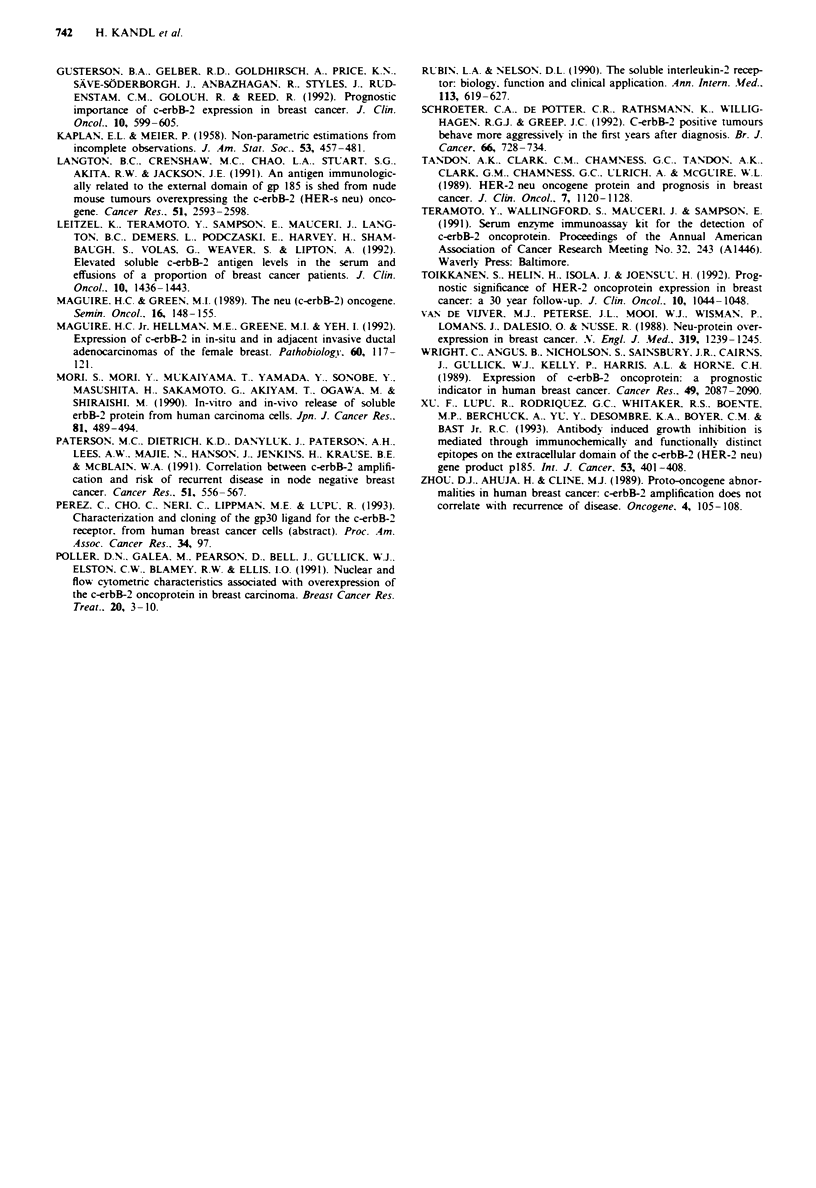# Soluble c-erbB-2 fragment in serum correlates with disease stage and predicts for shortened survival in patients with early-stage and advanced breast cancer.

**DOI:** 10.1038/bjc.1994.387

**Published:** 1994-10

**Authors:** H. Kandl, L. Seymour, W. R. Bezwoda

**Affiliations:** Department of Medicine, University of the Witwatersrand, Johannesburg, South Africa.

## Abstract

Seventy-nine patients with advanced breast cancer were tested for the presence, in serum, of a 110 kDa soluble, c-erbB-2 fragment. Thirty-nine patients were seropositive. There was no correlation between seropositivity and menopausal status, or with oestrogen status. In addition, no correlation could be found between tissue c-erbB-2 immunostaining for the external domain of the c-erbB-2 receptor and the presence of soluble c-erbB-2 in serum. The presence of serum soluble c-erbB-2, however, had a significant impact on survival of patients with advanced disease, suggesting that this test may become a useful independent prognostic indicator.


Br. J. Cancer (1994), 70, 739-742 © Macmillan Press Ltd., 1994

### Soluble c-*erb*B-2 fragment in serum correlates with disease stage and predicts for shortened survival in patients with early-stage and advanced breast cancer

H. Kandl, L. Seymour & W.R. Bezwoda

*Division of Clinical Haematology and Medical Oncology, Department of Medicine, University of the Witwatersrand, Johannesburg, South Africa.*

**Summary** Seventy-nine patients with advanced breast cancer were tested for the presence, in serum, of a 110 kDa soluble, c-*erb*B-2 fragment. Thirty-nine patients were seropositive. There was no correlation between seropositivity and menopausal status, or with oestrogen status. In addition, no correlation could be found between tissue c-*erb*B-2 immunostaining for the external domain of the c-*erb*B-2 receptor and the presence of soluble c-*erb*B-2 in serum. The presence of serum soluble c-*erb*B-2 , however, had a significant impact on survival of patients with advanced disease, suggesting that this test may become a useful independent prognostic indicator.

Breast cancer is a major health problem, affecting one in nine women in western countries. A particularly important goal is the early identification of poor-risk patients who may benefit from aggressive intervention with intensive chemotherapy.

While many tumour factors, including hormone receptor status, ploidy and growth fraction, and the expression of various oncogenes and proto-oncogenes by the tumour cells have been proposed as prognostic indicators, the results, to date, have been equivocal in a number of instances. Recent investigations into the role of amplification of the c-*erb*B-2 gene, the product of which is a transmembrane protein with extensive homology to the epidermal growth factor (EGF) receptor, have also appeared to give somewhat contradictory results. Gene amplification and increased c-*erb*B-2 expression have been reported in approximately 20% of patients with primary breast cancer (Clark & McGuire, 1991). Both gene amplification and increased expression of the gene product have been associated with a poorer prognosis in some studies. The discriminant power may, however, be confined to specific subsets of patients. Moreover, in a number of studies the prognostic significance of c-*erb*B-2 expression appears to be lost 5 or more years from diagnosis.

There has also been considerable interest, of late, in soluble forms of cell-surface receptors. Circulating soluble receptors include soluble forms of the insulin receptor and of the interleukin 2 receptor. Serum levels of soluble interleukin 2 receptor (IL-2R) can be shown to correlate with disease activity in autoimmune disorders (Rubin & Nelson, 1990) and with tumour bulk in certain lymphomas. A soluble, 100 kDa, c-*erb*B-2 fragment has been detected in the serum and effusions of patients with breast cancer (Mori *et al*., 1990; Leitzel *et al*., 1992 and may provide prognostic information in this disease.

We have undertaken a study of 79 patients with both early- and advanced-stage breast cancer in an attempt to evaluate the prognostic significance of elevations of serum soluble c-*erb*B-2.

### Materials and methods

### Methods

Blood samples were obtained from 79 patients attending the Breast Clinic of the Johannesburg Hospital between 1986 and 1993. Serum was stored at $-20°C$ until assay. Sampling was performed at the time of diagnosis of recurrent or metastatic disease. The 110 kDa, serum, soluble c-*erb*B-2 fragment was measured using a serum c-*erb*B-2, enzyme-linked immunoassay kit (Triton Diagnostics, Alameda, CA, USA). Briefly, monoclonal anti-c-*erb*B-2 antibody conjugates were added to aliquots of serum, incubated for 2 h, followed by addition of linking solution and then chromogen substrate. Absorbance was read in a spectrophotometer at 450 nm. Control and calibrator samples were run with each assay. Controls included samples from 24 healthy women falling into the same age range as the patients with breast cancer. The amount of c-*erb*B-2 protein was calculated from a standard curve. Results are expressed as units per ml of serum. Serum levels $\geqslant 10\ u\ ml^{-1}$ were deemed positive. This level was chosen as being two standard deviations above the mean for healthy women and was also the upper limit for the negative controls supplied with the kit. The antibody has no significant cross-reactivity with epidermal growth factor (EGF), and reacts only with the external domain of the c-*erb*B-2 molecule. Western blotting of samples with elevated levels confirmed the presence of a 100 kDa protein in serum which showed reactivity with this antibody.

Oestrogen receptor (ER) status and tissue c-*erb*B-2 were also determined when suitable specimens were available. ER was measured using the ERICA kit (Abbott Laboratories) method according to the manufacturer's instructions. Tissue c-*erb*B-2 determination was by means of immunohistochemistry using a monoclonal antibody to the external domain of c-*erb*B-2 (Triton Diagnostics) and a standard avidin-biotin procedure. Briefly, endogenous peroxidase was blocked using methanolic peroxide, and then blocking antibody, primary and control antibodies, secondary antibody, ABC (Vectastain) and diaminobenzidine (DAB) were layered on sequentially. Specimens were deemed positive if clear membrane immunostaining was observed. Suitable positive and negative controls were incorporated into each assay procedure.

### Statistical analysis

Disease-free survival, overall survival and survival from disease progression were analysed using SAS statistical software. Additional variables analysed included age, sex, site of disease, initial stage of disease, menopausal status and ER status (where available). Survival curves were generated using the method of Kaplan and Meier (1958), and were compared using the log-rank statistic.

### Ethical considerations

All patients gave informed consent prior to entry into the study. The study was approved by the Committee on Ethics

Correspondence: W.R. Bezwoda, Department of Medicine, University of the Witwatersrand Medical School, York Road, Parktown 2193 Johannesburg, South Africa.
Received 9 February 1994; and in revised form 6 May 1994.

of Human Research of the University of the Witwatersrand and was carried out in accordance with the principles of the Declaration of Helsinki.

### Results

### *Patient characteristics*

Forty-four out of 79 (56%) patients were post-menopausal at time of diagnosis. The mean age at presentation was 51.4 ± 13.1 (range 24–85) years. Further patient characteristics are shown in Tables I and II.

### *Serum soluble c-*erb*B-2*

Serum levels of the soluble fraction of c-*erb*B-2 ranged from 2 to 278 with a mean of 35 ± 57.6 u $ml^{-1}$. Intra- and inter-assay variation was <2%. Intra-patient variation of serum c-*erb*B-2 levels, when levels were tested in blood samples from 17 patients who had clinically stable disease and who had two or more separate blood samples taken at intervals of 14 to ⩽42 days, was also <2%.

Thirty-nine patients (49%) had serum soluble c-*erb*B-2 levels of ⩾10 u $ml^{-1}$. There was no correlation between the presence of elevated serum soluble c-*erb*B-2 level and menstrual status ($P = 0.66$). There was, however, a significant correlation between serum level and the type of treatment chosen for patients with stage IV disease [27 of 42 (64%) patients receiving chemotherapy were seropositive compared with 11 of 32 (34%) receiving hormonal therapy] (Table II). There was no correlation between the presence of raised serum soluble c-*erb*B-2 level and any specific site of relapse.

### *Tissue c-*erb*B-2 immunostaining*

Twenty-four patients had contemporaneous tumour tissue and serum samples available for c-*erb*B-2 determination. Tissue immunostaining did not correlate with the presence of soluble c-*erb*B-2 in serum (Table III). The presence of positive tissue immunostaining had no impact on overall survival, time to relapse or on survival from progression ($P = 0.26$).

### *Oestrogen receptor levels*

There was no significant correlation between oestrogen receptor expression among 37 patients of known receptor status and serum soluble c-*erb*B-2 levels ($P = 0.6$).

### *Survival and time to relapse*

The median overall survival of this cohort of patients from time of initial diagnosis was 44 ± 7.4 months (range 1–254 months). Among the 74 patients who either presented with or who had progressed to stage IV disease, the median survival time from progression was 19 ± 24.7 months (range 1–158). The presence of soluble c-*erb*B-2 fragment in serum at the time progression was diagnosed had a significant impact on overall survival of these patients. Seropositive patients had a median survival of 21 months *vs* 64 months for seronegative patients ($P = 0.03$) (Figure 1). The prognostic impact of soluble c-*erb*B-2 on survival was lost when the analysis was confined to ER-positive patients, possibly because of the low number of such patients.

**Figure 1** Influence of the presence of soluble c-*erb*B-2 fragment in the serum on prognosis of patients with breast cancer. Overall survival. ●, Patients with serum soluble c-*erb*B02 <10 u $ml^{-1}$; O, patients with serum soluble c-*erb*B-2 ⩾10 u $ml^{-1}$.

**Table I** Serum soluble c-*erb*B-2: patient characteristics and seropositivity rate

| | Patient characteristics | | Soluble c-erbB-2 | | | | |
|---|---|---|---|---|---|---|---|
| | | | Positive | | Negative | | |
| | *Number* | *(%)* | *Number* | *(%)* | *Number* | *(%)* | *P-value* |
| Menopausal status | | | | | | | |
| Pre | 35 | (44) | 17 | (49) | 18 | (51) | NS |
| Post | 44 | (56) | 22 | (50) | 22 | (50) | |
| Stage at presentation | | | | | | | |
| I | 4 | (5) | 1 | (25) | 3 | (75) | |
| II | 31 | (39) | 11 | (35) | 20 | (65) | |
| III | 19 | (24) | 10 | (53) | 9 | (47) | |
| IV | 25 | (32) | 17 | (68) | 8 | (32) | |
| Stage at sampling | | | | | | | |
| IV | 74 | (94) | 39 | (49) | 40 | (51) | |
| ER status | | | | | | | |
| Positive | 15 | (20) | 7 | (47) | 8 | (53) | |
| Negative | 25 | (33) | 12 | (48) | 13 | (52) | NS |
| Unknown | 39 | (47) | 20 | (51) | 19 | (49) | |

NS, not significant.

**Table III** Breast tumour tissue expresson of c-*erb*B-2 protein and the presence of serum soluble c-*erb*B-2 fragment

| | *Serum soluble c-erbB-2 positive* | *Serum soluble c-erbB-2 negative* |
|---|---|---|
| Tissue immunostaining c-*erb*B-2 | | |
| positive | 6 | 4 |
| negative | 6 | 8 |

**Table II** Serum soluble c-*erb*B-2: patient characteristics and response to therapy for stage IV disease

| | | | Soluble c-erbB-2 | | | | |
|---|---|---|---|---|---|---|---|
| | | | Positive | | Negative | | |
| | *Number* | *(%)* | *Number* | *(%)* | *Number* | *(%)* | *P-value* |
| Treatment for stage IV disease | | | | | | | |
| Hormonal | 32 | (42) | 11 | (34) | 21 | (66) | $P<0.05$ |
| Chemotherapy | 42 | (48) | 27 | (64) | 15 | (34) | |
| Response to first-line therapy | | | | | | | |
| Complete and partial response | 40 | (52) | 20 | (50) | 20 | (50) | NS |
| No response | 34 | (43) | 18 | (53) | 16 | (47) | |

NS, not significant.

*Response to therapy*

Serum soluble c-*erb*B-2 had no influence on response to either initial ($P = 0.64$) or salvage treatment for stage IV disease ($P = 0.78$).

### Discussion

The c-*erb*B-2 protein is a 185 kDa transmembrane protein with tyrosine kinase activity. It comprises both an extracellular and an intracellular domain. While the extracellular domain has ligand-binding activity, the ligand has yet to be clearly defined (Maguire & Green, 1989), but is thought to act as a growth factor (Perez *et al.*, 1993). Antibodies to c-*erb*B-2 have been shown to inhibit both anchorage-dependent and anchorage-independent growth *in vivo* (Xu *et al.*, 1993).

c-*erb*B-2 has been found to be amplified in 20–30% of primary breast cancers, and gene amplification correlates with oncoprotein overexpression. c-*erb*B-2's impact on prognosis is, however, somewhat controversial. A number of investigators have reported a correlation between c-*erb*B-2 amplification and survival in node-positive primary breast cancer (Tandon *et al.*, 1989; Borg *et al.*, 1990). However, both Zhou *et al.* (1989) and Toikkanen *et al.* (1992) failed to demonstrate any impact of c-*erb*B-2 expression on survival in node-positive patients. Conflicting results have also been reported in node-negative patients (Wright *et al.*, 1989; Paterson *et al.*, 1991). Allred *et al.* (1992) found a highly significant correlation between disease-free survival and c-*erb*B-2 expression, but only in specific subsets of patients (small tumour size, ER positive and no significant *in situ* component), so-called 'low-risk patients'. Furthermore, while Gusterson *et al.* (1992) found c-*erb*B-2 immunostaining to have an overall prognostic impact only in patients with node-positive disease, c-*erb*B-2-positive, node-negative patients receiving adjuvant chemotherapy fared less well in their study than those who were c-*erb*B-2 negative. These findings tended to suggest that, whatever influence the presence of c-*erb*B-2 expression has on the biology of breast cancer, this effect is confined to the earlier clinical phases of the illness.

In addition, it has been suggested that c-*erb*B-2 amplification and protein expression correlate both with poor histological grade and lack of ER expression (Cline *et al.*, 1987; Schroeter *et al.*, 1992). Poller *et al.* (1991) demonstrated that overexpression of c-*erb*B-2 is significantly correlated with S-phase and proliferative index in ductal carcinoma *in situ* ($P = 0.001$), as well as in early invasive duct carcinoma ($P = 0.04$).

There is also evidence to suggest that c-*erb*B-2 overexpression may be preferentially associated with certain histological subtypes of breast cancer. Van de Vijver *et al.* (1988) described a high incidence of c-*erb*B-2 overexpression in large-cell, comedo-type ductal carcinoma *in situ* as compared with invasive ductal carcinoma, suggesting either that the invasive ductal carcinomas that are c-*erb*B-2 positive are derived from a specific type (large-cell comedo) of ductal carcinoma *in situ* or that c-*erb*B-2 expression may be lost during tumour invasion and progression. Evidence supporting the first theory is provided by Maguire *et al.* (1992), who found that while tumours with c-*erb*B-2-negative *in situ* components had immunonegative invasive components, tumours with immunopositive comedo-type *in situ* components had immunopositive invasive ductal carcinoma. It should be pointed out, however, that the immunostaining was frequently more intense in the *in situ* components than in the invasive carcinomas.

Soluble forms of the c-*erb*B-2 protein have been reported in the serum of patients with breast cancer, and in addition have been shown to correlate with disease bulk as well as with tissue overexpression in an animal model (Langton *et al.*, 1991). No reports have, however, been published to date on the prognostic significance of soluble c-*erb*B-2 in patients with breast cancer.

The present study examined a group of 79 women with advanced breast cancer. The method used in this study measured a 100 kDa c-*erb*B-2 antigen fragment, which is not detected in the serum of normal controls or in patients with benign breast disease (Teramoto *et al.*, 1991). A surprisingly high frequency of elevated serum soluble c-*erb*B-2 levels was found, possibly because this study included mainly patients with aggressive disease, with 25 patients presenting with advanced breast cancer and all but five of the remainder having progressed to stage IV disease. In addition, there was a statistically significantly higher incidence of seropositivity in patients given chemotherapy as first-line therapy for progressive disease – indicative of the perception that these patients were suffering from aggressive disease. In addition, the presence of serum soluble c-*erb*B-2 fragment concentrations of $>10\,u\,ml^{-1}$ had a significant impact on overall survival from diagnosis of metastatic disease.

The lack of correlation between seropositivity and tissue expression of c-*erb*B-2 raises some interesting possibilities. While the lack of correlation may be due to low sample number, definite tissue staining was demonstrated in 10 of the 24 patients with tumour samples available for examination. This frequency was, again, a relatively high rate of c-*erb*B-2 expression. While it may be argued that lack of tissue immunostaining is related to the sensitivity of the method, both tissue-positive and serum-ngetaive as well as tissue-negative and serum-positive cases were found. Among the serum soluble c-*erb*B-2-negative patients with negative tissue expression there were three patients with extremely high serum levels (range 120–160 $u\,ml^{-1}$) and with extensive disease, while all four patients with negative serum soluble c-*erb*B-2 tissue and positive tissue staining demonstrated strong positive staining and also had extensive disease. Since both the serum and tissue assays were performed using a monoclonal antibody specific for the external domain of c-*erb*B-2, these findings suggest the possibility that loss of tissue expression may result from proteolytic cleavage, with release of the external domain and transfer into the blood, rendering the tissue negative to reaction with the antibody used, or that the serum component represents an alternatively spliced variant lacking the membrane domain. This question will be addressed in future studies by using antibodies to both the internal domain as well as to the external domain of the c-*erb*B-2 molecule.

Whatever the pathophysiological explanation, assay for soluble c-*erb*B-2 in serum is a relatively simple test, requiring only a blood sample rather than tissue. Soluble c-*erb*B-2 may offer prognostic information with seropositivity being a predictor of shorter survival in patients with breast cancer.

### References

ALLRED, D.C., CLARK, G.M., TANDON, A.K., MOLINA, R., TORMEY, D.C., OSBORNE, C.K., GILCHRIST, K.W., MANSOUR, E.G., ABELOFF, M., EUDY, L. & MCGUIRE, W.L. (1992). HER-2 neu in node negative breast cancer: prognostic significance of overexpression influenced by the presence of in-situ carcinoma. *J. Clin. Oncol.*, **10**, 1049–1056.

BORG, A., TANDON, A.K., SIGURDSSON, H., CLARK, G.M., FERNÖ, M., FUQUA, S.A., KILLANDER, D. & MCGUIRE, W.L. (1990). HER-2 neu amplification predicts poor survival in node-positive breast cancer. *Cancer Res.*, **50**, 4332–4337.

CLARK, G.M. & MCGUIRE, W.L. (1991). Follow-up study of HER-2 neu amplification in primary breast cancer. *Cancer Res.*, **51**, 944–948.

CLINE, M.J., BATTINFORA, H. & YOKOTA, J. (1987). Proto-oncogene abnormalities in human breast cancer: correlation with anatomic features and clinical course of disease. *J. Clin. Oncol.*, **7**, 999.

GUSTERSON, B.A., GELBER, R.D., GOLDHIRSCH, A., PRICE, K.N., SÄVE-SÖDERBORGH, J., ANBAZHAGAN, R., STYLES, J., RUDENSTAM, C.M., GOLOUH, R. & REED, R. (1992). Prognostic importance of c-erbB-2 expression in breast cancer. *J. Clin. Oncol.*, **10**, 599-605.

KAPLAN, E.L. & MEIER, P. (1958). Non-parametric estimations from incomplete observations. *J. Am. Stat. Soc.*, **53**, 457-481.

LANGTON, B.C., CRENSHAW, M.C., CHAO, L.A., STUART, S.G., AKITA, R.W. & JACKSON, J.E. (1991). An antigen immunologically related to the external domain of gp 185 is shed from nude mouse tumours overexpressing the c-erbB-2 (HER-s neu) oncogene. *Cancer Res.*, **51**, 2593-2598.

LEITZEL, K., TERAMOTO, Y., SAMPSON, E., MAUCERI, J., LANGTON, B.C., DEMERS, L., PODCZASKI, E., HARVEY, H., SHAMBAUGH, S., VOLAS, G., WEAVER, S. & LIPTON, A. (1992). Elevated soluble c-erbB-2 antigen levels in the serum and effusions of a proportion of breast cancer patients. *J. Clin. Oncol.*, **10**, 1436-1443.

MAGUIRE, H.C. & GREEN, M.I. (1989). The neu (c-erbB-2) oncogene. *Semin. Oncol.*, **16**, 148-155.

MAGUIRE, H.C. Jr, HELLMAN, M.E., GREENE, M.I. & YEH, I. (1992). Expression of c-erbB-2 in in-situ and in adjacent invasive ductal adenocarcinomas of the female breast. *Pathobiology*, **60**, 117-121.

MORI, S., MORI, Y., MUKAIYAMA, T., YAMADA, Y., SONOBE, Y., MASUSHITA, H., SAKAMOTO, G., AKIYAM, T., OGAWA, M. & SHIRAISHI, M. (1990). In-vitro and in-vivo release of soluble erbB-2 protein from human carcinoma cells. *Jpn. J. Cancer Res.*, **81**, 489-494.

PATERSON, M.C., DIETRICH, K.D., DANYLUK, J., PATERSON, A.H., LEES, A.W., MAJIE, N., HANSON, J., JENKINS, H., KRAUSE, B.E. & MCBLAIN, W.A. (1991). Correlation between c-erbB-2 amplification and risk of recurrent disease in node negative breast cancer. *Cancer Res.*, **51**, 556-567.

PEREZ, C., CHO, C., NERI, C., LIPPMAN, M.E. & LUPU, R. (1993). Characterization and cloning of the gp30 ligand for the c-erbB-2 receptor, from human breast cancer cells (abstract). *Proc. Am. Assoc. Cancer Res.*, **34**, 97.

POLLER, D.N., GALEA, M., PEARSON, D., BELL, J., GULLICK, W.J., ELSTON, C.W., BLAMEY, R.W. & ELLIS, I.O. (1991). Nuclear and flow cytometric characteristics associated with overexpression of the c-erbB-2 oncoprotein in breast carcinoma. *Breast Cancer Res. Treat.*, **20**, 3-10.

RUBIN, L.A. & NELSON, D.L. (1990). The soluble interleukin-2 receptor: biology, function and clinical application. *Ann. Intern. Med.*, **113**, 619-627.

SCHROETER, C.A., DE POTTER, C.R., RATHSMANN, K., WILLIGHAGEN, R.G.J. & GREEP, J.C. (1992). C-erbB-2 positive tumours behave more aggressively in the first years after diagnosis. *Br. J. Cancer*, **66**, 728-734.

TANDON, A.K., CLARK, C.M., CHAMNESS, G.C., TANDON, A.K., CLARK, G.M., CHAMNESS, G.C., ULRICH, A. & MCGUIRE, W.L. (1989). HER-2 neu oncogene protein and prognosis in breast cancer. *J. Clin. Oncol.*, **7**, 1120-1128.

TERAMOTO, Y., WALLINGFORD, S., MAUCERI, J. & SAMPSON, E. (1991). Serum enzyme immunoassay kit for the detection of c-erbB-2 oncoprotein. Proceedings of the Annual American Association of Cancer Research Meeting No. 32, 243 (A1446). Waverly Press: Baltimore.

TOIKKANEN, S., HELIN, H., ISOLA, J. & JOENSUU, H. (1992). Prognostic significance of HER-2 oncoprotein expression in breast cancer: a 30 year follow-up. *J. Clin. Oncol.*, **10**, 1044-1048.

VAN DE VIJVER, M.J., PETERSE, J.L., MOOI, W.J., WISMAN, P., LOMANS, J., DALESIO, O. & NUSSE, R. (1988). Neu-protein overexpression in breast cancer. *N. Engl. J. Med.*, **319**, 1239-1245.

WRIGHT, C., ANGUS, B., NICHOLSON, S., SAINSBURY, J.R., CAIRNS, J., GULLICK, W.J., KELLY, P., HARRIS, A.L. & HORNE, C.H. (1989). Expression of c-erbB-2 oncoprotein: a prognostic indicator in human breast cancer. *Cancer Res.*, **49**, 2087-2090.

XU, F., LUPU, R., RODRIQUEZ, G.C., WHITAKER, R.S., BOENTE, M.P., BERCHUCK, A., YU, Y., DESOMBRE, K.A., BOYER, C.M. & BAST Jr, R.C. (1993). Antibody induced growth inhibition is mediated through immunochemically and functionally distinct epitopes on the extracellular domain of the c-erbB-2 (HER-2 neu) gene product p185. *Int. J. Cancer*, **53**, 401-408.

ZHOU, D.J., AHUJA, H. & CLINE, M.J. (1989). Proto-oncogene abnormalities in human breast cancer: c-erbB-2 amplification does not correlate with recurrence of disease. *Oncogene*, **4**, 105-108.